# Role of Metabolic Endotoxemia in Systemic Inflammation and Potential Interventions

**DOI:** 10.3389/fimmu.2020.594150

**Published:** 2021-01-11

**Authors:** Shireen Mohammad, Christoph Thiemermann

**Affiliations:** William Harvey Research Institute, Queen Mary University of London, London, United Kingdom

**Keywords:** metabolic endotoxemia, lipopolysaccharide, high-fat diet, Toll-like receptor, antimicrobial peptides, gut permeability

## Abstract

Diet-induced metabolic endotoxemia is an important factor in the development of many chronic diseases in animals and man. The gut epithelium is an efficient barrier that prevents the absorption of liposaccharide (LPS). Structural changes to the intestinal epithelium in response to dietary alterations allow LPS to enter the bloodstream, resulting in an increase in the plasma levels of LPS (termed metabolic endotoxemia). LPS activates Toll-like receptor-4 (TLR4) leading to the production of numerous pro-inflammatory cytokines and, hence, low-grade systemic inflammation. Thus, metabolic endotoxemia can lead to several chronic inflammatory conditions. Obesity, diabetes, and non-alcoholic fatty liver disease (NAFLD) can also cause an increase in gut permeability and potential pharmacological and dietary interventions could be used to reduce the chronic low-grade inflammation associated with endotoxemia.

## Introduction

Cani and colleagues (1) first defined metabolic endotoxemia as a diet-induced, 2–3-fold increase in plasma LPS levels associated with low-grade inflammation, ultimately leading to the development of cardiometabolic diseases. The serum levels of LPS seen after a 4-week exposure to high-fat diet (HFD) were similar to that of mice continuously infused with LPS for 4 weeks to induce metabolic endotoxemia (1). It should, however, be noted that the levels of LPS observed after the above diet (and, indeed, in other models of metabolic endotoxemia) are 10–15 times lower compared to those seen in animals or man with sepsis (1). Inflammation is a normal process of the host defense. However, an unresolved (chronic) inflammatory response leads to low-grade, systemic inflammation (2). Indeed, metabolic endotoxemia causes a state of low-grade inflammation, which is a pathological feature of a range of chronic conditions including type 2 diabetes mellitus (T2DM), non-alcoholic fatty liver disease (NAFLD), chronic kidney disease, and atherosclerosis (2).

## Immune Activation in Metabolic Endotoxemia

### LPS

**Figure 1:**
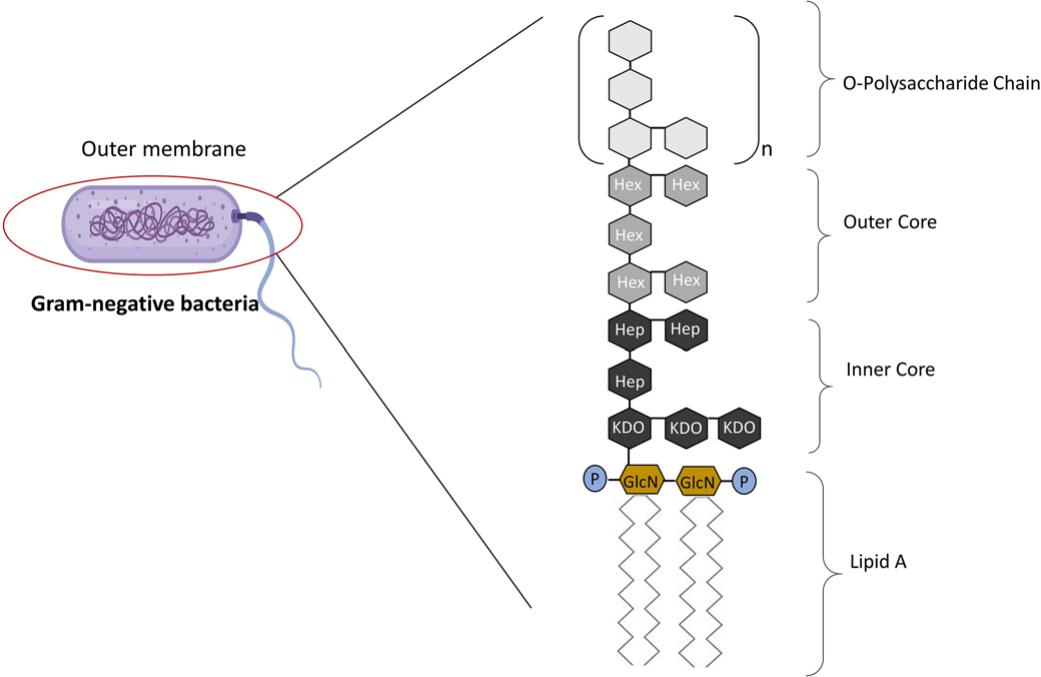
The Structure of LPS. LPS is made up of three parts: Lipid A, core, and the O-polysaccharide chain. Covalently attached to lipid A is the core part of the molecule, divided into the inner and outer core. The inner core is next to the lipid A section, which contains sugars such as L-glycerol-D-manno heptose (Hep) and 3-deoxy-D-manno-octulosonic acid (Kdo). The outer core contains common sugars such as hexosamines and hexoses (Hex). Attached to the outer core are repeating subunits of oligosaccharides, referred to as the O-chain (3). Adapted from Erridge et al. (3).

The term “lipopolysaccharide” was first introduced when it became apparent that endotoxin contains both carbohydrates and lipids (3). Endotoxins are complexes made up of LPS that form the major component of the outer wall of Gram-negative bacteria, while exotoxins are defined as those toxins that are actively secreted by bacteria with a conserved lipid A “endotoxic” component (4, 5). LPS is composed of three parts: Lipid A (fatty acid and hydrophobic tail); the core divided into the inner and outer core (oligosaccharide containing sugar residues) and the O side chain (repeating sugar residues) (**Figure 1**) (6). LPS is a potent activator of the inflammatory response and is a crucial pathogen-associated molecular patterns (PAMPs) in Gram-negative bacteria, consequently small amounts of LPS present in the blood due to a bacterial infection is sufficient enough to elicit an inflammatory response through the interaction with Toll-like receptors (TLRs) (7). Hence, LPS is responsible for pathophysiologic symptoms which is characteristic of infection (8, 9). The ability of LPS to evoke signaling events and trigger the release of cytokines from macrophages (and, indeed of Kupffer cells in the liver) is dependent on the type of lipid A component within the LPS structure.

### Toll-Like Receptors

TLRs are single domain transmembrane plasma bound receptors that belong to the pattern recognition receptors (PRRs) family as well as NOD-like receptors (NLRs) and mannose. They are important receptors in the innate immune system, as they are able to detect the presence of microbial infections (10). TLRs are expressed by immune cells such as macrophages, dendritic cells, and non-immune cells, recognizing a variety of stimuli, including conserved motifs of microbes known as PAMPs derived from various microbes such as LPS, lipopeptides, viral double-stranded RNA, bacterial DNA, and danger-associated molecular pattern molecules (DAMPs) derived from damaged cells, such as heat shock proteins to help initiate and promote the immune response (11). Each TLR recognizes specific microbial components derived from pathogens (**Table 1**) and upon the recognition of PAMPs, TLRs initiate the proinflammatory signaling pathway *via* the recruitment of specific adaptor molecules and the activation of transcription factors, nuclear factor-κB (NF-κB), and interferon-regulatory factors (IRFs) resulting in an immune response by producing inflammatory cytokines, type I interferon (IFN) and other mediators. Pro-inflammatory cytokines and the activation of microbicidal responses is required to eliminate infection, however overactivation of PRRs can cause systemic inflammatory responses (15).

**Table 1:** Examples of pattern recognition receptors (PRRs) and their specific pathogen-associated molecular patters (PAMPs).

TLR	Origin of PAMP	Bacterial PAMPs
TLR1/TLR2	Gram-positive and Gram-negative bacteria	Triacyl lipopeptides
TLR2	Gram-positive and Gram-negative bacteria Gram-positive bacteria *Neisseria* spp. Fungi Fungi Virus	Peptidoglycan Lipoteichoic acid Diacyl lipopeptides Zymosan β-Glycan Envelope glycoproteins
TLR2/TLR6	*Saccharomyces cerevisiae* Streptococcus Bacteria (Mycoplasma)	Zymosan Lipoteichoic acid Diacyl lipopeptides
TLR3	Double-stranded RNA virus	dsRNA
TLR4	Gram-negative bacteria *Candida albicans* *Candida albicans* *Candida albicans* Host	LPS-MD2 Mannan Phospholipomannan O-linked mannosyl residues Hyaluronan
TLR5	Flagellated bacteria	Flagellin
TLR7/8	RNA viruses	ssRNA
TLR9	Bacteria Virus Protozoa	cpG DNA DNA Hemozoin
TLR11	Parasites	Profilin-like molecules

Table adapted from Marshall et al. (12), Doughty (13), and Kawai et al. (14).

The Toll receptor was first discovered for its role in the embryonic development of fruit-fly *Drosophila melanogaster*, as a mutation in the Toll gene caused abnormal development in the embryos compared to wild-type flies and they were more susceptible to fungal infection. These mutated flies were termed Toll, the German word for “wow” (16–18). Ten TLRs members have been identified in humans (TLR1–TLR10) and 12 in mice (TLR1–TLR9, TLR11–TLR13). TLRs can be divided into two subpopulations in terms of their cellular localization found either on the outer membrane (TLR1, 2, 4, 5, 6, 10) capable of recognizing microbial membrane components such as lipoprotein, protein, and lipids or found in intracellular vesicles such as the endosomal, lysosomal membranes, or the endoplasmic reticulum (TLR3, 7, 9) that target viruses that enter *via* endocytosis (19, 20). TLRs are able to recognize their respective PAMPs, as each TLR is composed of an ectodomain with leucine-rich repeats (LRRs) that adopts a horseshoe shaped structure connected with the cytosolic carboxy-terminal domain, the cytoplasmic Toll/IL-IR (TIR) domain and a transmembrane domain that is required for initiating the downstream signaling pathway (15). The LRR motif is responsible for the recognition of their respective pathogen and the cytosolic TIR domain is necessary for the interaction with adaptor proteins, including myeloid differentiation primary response gene 88 (MyD88), TIR-containing adaptor protein (TIRAP), TRIF-related adaptor molecule (TRAM), and Toll/interleukin-1 receptor domain-containing adapter inducing interferon-β (TRIF) and hence necessary for the initiation of the downstream signaling transduction (21).

### Toll-Like Receptor 4 Signaling

TLR4 recognizes bacterial LPS, binding of which leads to cellular activation, causing the release of proinflammatory cytokines. This strategy of recognition is the first line of defense against bacterial infections and LPS is the most powerful immunostimulant known to date. The lipid A moiety is the main PAMP within LPS and if excessive signaling occurs through TLR4, this can induce systemic inflammation, a cytokine-storm, and ultimately sepsis (22).

On the cell surface, the first protein involved in the recognition of LPS is the LPS-binding protein (LBP). LBP is present as a soluble protein or a plasma membrane protein in the bloodstream, it recognizes and forms a complex with the lipid A part of LPS in the plasma allowing LPS to dock at the LPS receptor complex resulting in the LPS-LBP complex. LBP allows LPS to interact with CD14 on the cell surface, a glycosylphoshatidylinositol-anchored protein containing LRRs, binds LBP and transfers LPS-LBP to the co-receptor of TLR4, myeloid differentiation protein-2 (MD-2) and TLRs are able to detect LPS with the help of this accessory protein, MD-2, a secreted glycoprotein, which non-covalently associates with TLR4 and acts as a binding site for LPS (4). Once LPS binds to the TLR-CD14-MD-2 complex and is recognized, TLR4 is activated and undergoes oligomerization, allowing the recruitment of the downstream adaptors through the interaction with TIR domains due to the conformational change of the extracellular domain allowing the dimerization of the cytoplasmic TIR domain. The LPS/TLR4 mediated response triggers two distinct signaling pathways: the MyD88-dependent pathway which tends to occur earlier and is utilized by all TLRs, except TLR3. This involves the recruitment of TIRAP at the cell membrane followed by the recruitment of MyD88 and the MyD88-independent pathway which occurs during the late phase response which involves the trafficking of TLR4 to the endosome and the recruitment of TRAM and TRIF (22, 23).

### MyD88-Dependent Pathway

**Figure 2:**
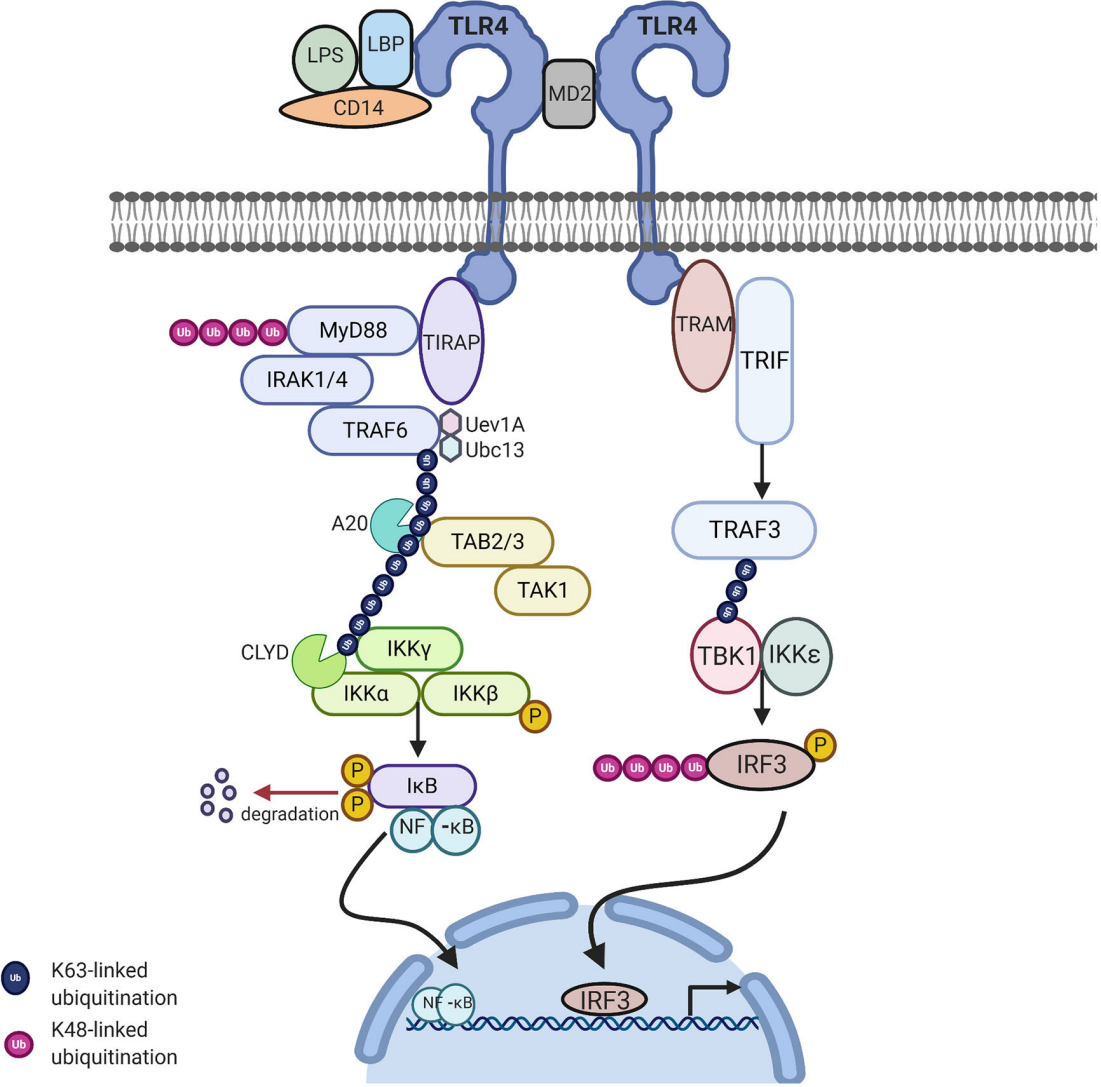
TLR4 signaling *via* MyD88-dependent and independent pathway to activate NFkB related target genes: Upon stimulation of myeloid differentiation primary response protein 88 (MyD88) dependent pathway involves the activation of MyD88 which recruits IL-1 receptor-associated kinase-4 (IRAK-4). IRAK-4 phosphorylates IRAK-1 and allows tumor necrosis factor receptor associated factor 6 (TRAF6) to associate with IRAK1. IRAK1/TRAF6 then activates TAK_1_, TAB_1_, and TAB_2_. The TRAF6, TAK_1_, TAB_1_, and TAB_2_ forms a larger complex with ubiquitin-conjugating enzyme E32 variant 1 isoform A (Ubc13) and Uev1A which activates TAK_1_. Polyubiquitin chain is then removed by A20 and conserved cylindromatosis (CYLD). Activated TAK_1_ phosphorylates the IKK complex (IKKα. IKKβ and IKKγ) ultimately resulting in the translocation of nuclear factor-κB (NF-κB) into the nucleus, resulting in the transcription of proinflammatory cytokines. MyD88 independent pathway involves TIR-domain-containing adapter-inducing interferon-β (TRIF) leading to the activation of TNF receptor associated factor 3 (TRAF3) and the translocation of interferon regulatory factor 3 (IRF3) to the nucleus leading to IFNB gene transcription. Image made using BioRender.

MyD88 belongs to the family of cytosolic TIR domain-containing adaptor molecules (24). Upon stimulation with a ligand, the MyD88-dependent pathway involves the recruitment and activation of IL-1 receptor-associated kinase 4 (IRAK-4), linking TLRs to IL-1Rs. The death domain recognizes the conformational change of the TIR domain of TLRs and binds to the new conformation allowing the transfer of signaling by the interaction of the death domain of the MyD88 with IRAK kinases family membranes, known as Myddosome. There are four IRAKs (IRAK-1, 2, 3, 4) that contain an N-terminal death domain and a central serine/threonine-kinases domain. During the Myddosome formation, IRAK4 activates IRAK1 and undergoes autophosphorylation, allowing the association with the adaptor protein, TNF-α receptor associated factor 6 (TRAF6), which is critical for the activation of this pathway. The activation of IRAK1 allows the binding to the TRAF domain of TRAF6. The IRAK1/TRAF6 complex then dissociates from the receptor and associates with TAK_1_ and TAK_1_-binding proteins (TAB_1_ and TAB_2_). TAK_1_ is a member of the mitogen activated protein kinase kinase kinase (MAPKKK) family and forms a complex with the regulatory subunits (TAB_1_ and TAB_2_) that interact with K63-linked polyubiquitin chains generated by TRAF6, along with ubiquitin-conjugating enzyme, ubiquitin-conjugating enzyme E32 variant 1 isoform A (UBC_13_) and UEV_1_A to drive the activation of the transforming growth factor-β-activated kinase (TAK1). The TRAF6, TAK_1_, TAB_1_, and TAB_2_ complex then moves to the cytoplasm and forms a complex with UBC_13_. TAK1 then activates the IKK complex, the catalytic subunits IKKα and IKKβ and the regulatory subunit NEMO (known as IKKγ). Through ubiquitin chains TAK_1_ phosphorylates IKKβ. This leads to the degradation of IKβ and the release of free NF-κB (p50/p65). The IKK complex phosphorylates the NF-κB inhibitory protein, IκBα, which in turn allows the translocation of NF-κB to the nucleus, initiating the transcription of proinflammatory genes including IL-18, IL-6, IL-1α, and IL-1β (**Figure 2**) (15).

### MyD88-Independent Pathway

The MyD88-independent pathway is also known as the TRIF dependent pathway. This pathway is initiated by TRAM and TRIF. This pathway is specific to a few TLRs and once stimulated the adapter protein, TRIF is important for recruiting TNF receptor associated factor 3 (TRAF3). TRAF3 recruits the IKK-related kinases, TRAF family member-associated NF-κB activator-binding kinase (TBK1) and IKKε, leading to the phosphorylation of interferon regulatory factor 3 (IRF3), which forms a dimer and results in the translocation into the nucleus, resulting in the activation of the expression of IFN inducible genes and inflammatory mediators (**Figure 2**) (15, 25).

## Intestinal Change

### Components of the Intestinal Epithelial

**Figure 3:**
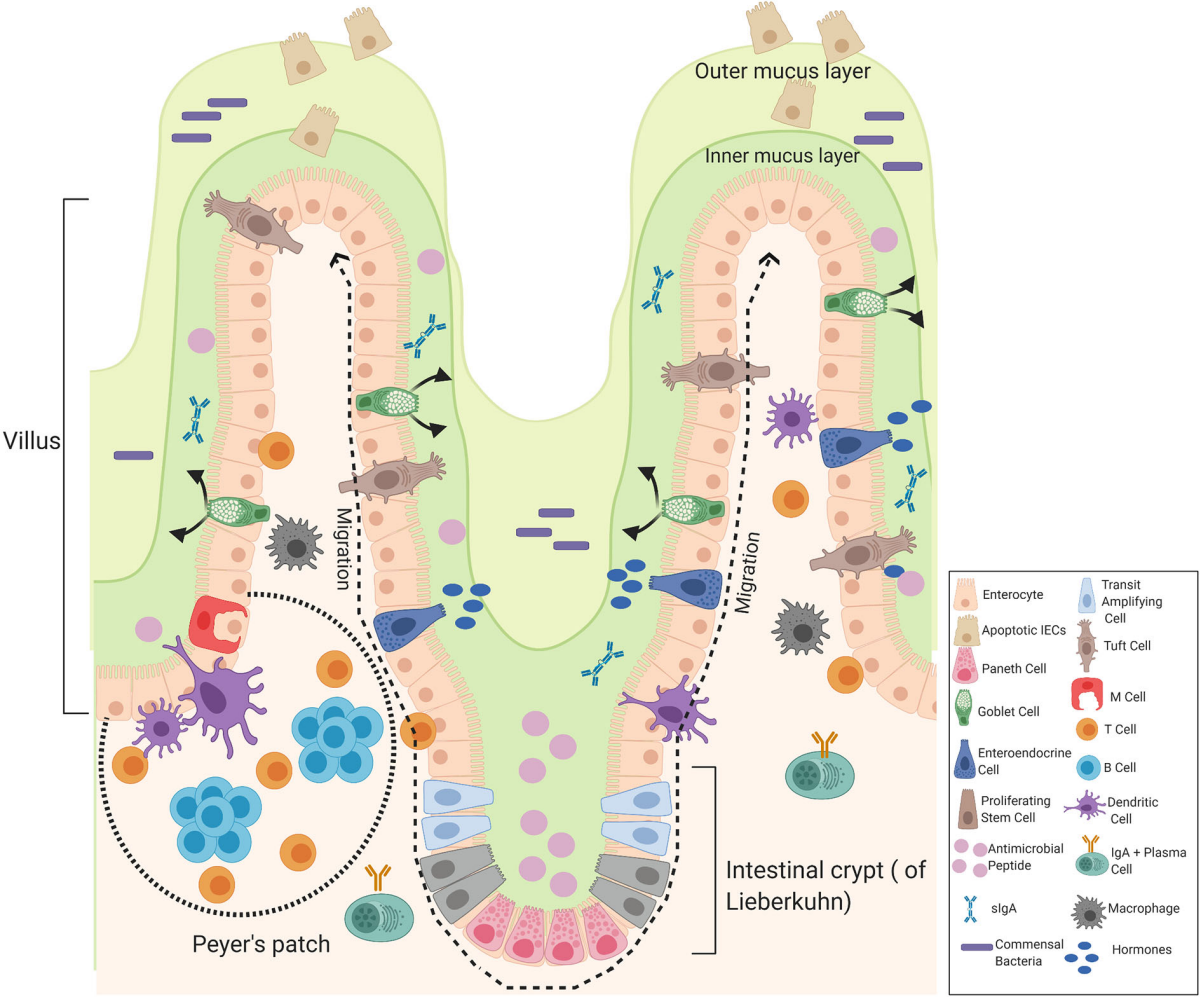
Components of the intestinal epithelial cells of the small intestine. The intestinal epithelium is composed of a signal layer of intestinal epithelial cells (IECs) covered by a mucus layer continuously secreted by goblet cells to act as a first physical barrier against pathogenic bacteria. The outer mucus layer is colonized by bacteria whereas the inner mucus is a bacteria-free loose layer. Paneth cell secrete antimicrobial peptides (AMPs) into the inner mucus and enteroendocrine cells produce hormones where secretory IgA (sIgA) are also present to protect against commensal bacteria and contribute to the formation of a biochemical barrier. Stem cells are located at the base of the crypt that give rise to four cells in the intestinal epithelial: enterocytes, enteroendocrine cells, goblet cells, and Paneth cells that migrate upwards and move to the distal end of a villus where they are shed. Enteroendocrine cells produce hormones, goblet cells produce mucus, and Paneth cells produce AMPs. Other cells include truft cells, macrophages, dendritic cells T-cells, B-cells, and M-cells in the Peyer’s patches of the intestine involved in transporting antigens from the lumen to cells of the immune system. Image made using BioRender.

The small and large intestines form the major part of the gastrointestinal tract that follows the stomach. The small intestine consists of three divisions: the duodenum, the jejunum, and the ileum. The duodenum is the first and shortest part of the small intestine involved in chemical digestion. The jejunum is the middle part of the small intestine where nutrients are absorbed into the blood and the ileum is between the jejunum and the cecum. The mucous membrane lining the jejunum is covered with villi and the ileum is where the remaining nutrients are absorbed, the inner surface is also covered with villi. The small intestine consists of four tissue layers: mucosa, submucosa, muscularis, and the serosa. The intestinal epithelial are one-cell-thick, lining of the surface of the intestinal epithelium that form a barrier between the mucosal tissue and the lumen to protect humans (and animals) against invading microorganisms. Each epithelium has a finger-like projection called villi, brush border, crypt, and basolateral plasma membrane structure (26, 27). Each individual villus contains a rich capillary network and between the villi are intestinal glands (crypts of Lieberkuhn) and each crypt contains approximately 250 cells with the size and organization of the crypt being generally uniform (28). Stem cells located at the base of the crypt give rise to four cells in the intestinal epithelial including enterocytes, enteroendocrine cells, goblet cells, and Paneth cells which are only found in the small intestine, these differentiated cells are continuously being supplied and are pushed upward towards the mouth of the crypt where they move to the distal end of a villus and are shed. Hence, it is the most rapid self-renewing tissue of adult mammals (27). The enterocytes are the most abundant cells and predominantly line the lumen of the small intestine that are important for digestion and absorption of nutrients. The enterocytes are assisted by the mucus secreting goblet cells found in large numbers on villi important for the synthesis and secretion of mucin. The mucus layer is the first line of defense, as it is a chemical barrier, consisting of digestive secretions, immune molecules, antimicrobial peptides (AMPs), and cytokines. This layer is crucial to limit the contact between the microbiome and epithelial cells (29). The gastrointestinal hormone secreting enteroendocrine cells are important for hormonal regulation and Paneth cells are important for the immunological function, secreting antimicrobial peptides to promote the exclusion of bacteria from the epithelial surface. Paneth cells are found at the base of the small intestine crypts, hence protecting the stem cells (30). Other components of the intestinal epithelial include the luminal release of secretory IgA and the microfold cells (M cells), important for the delivery of antigens to immune cells. Tuft cells which secrete endorphins and enzymes such as prostaglandins. Immune cells are also present, including T cells, B cells, dendritic cells, and macrophages that help to maintain intestinal homeostasis (**Figure 3**) (29).

### Cell Junctional Protein Complexes and a Leaky Intestinal Barrier

**Figure 4:**
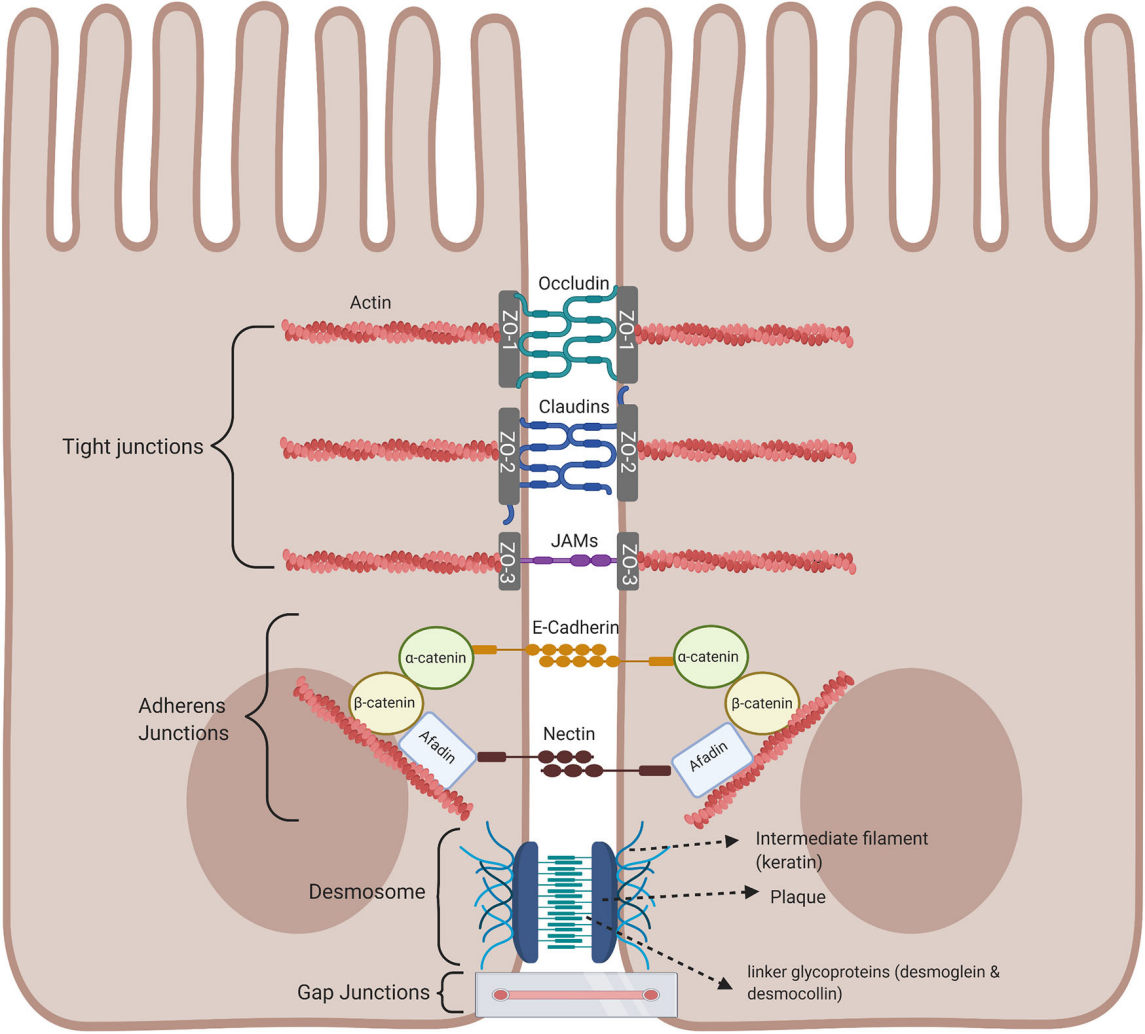
Intestinal epithelial cell junctional proteins. Tight junctions are at the apical end of junctional complexes composed of three transmembrane proteins: occludin claudins and junctional adhesion molecules (JAMs) that bind intracellular membrane proteins, zonula occludens (ZOs) which connects the transmembrane tight junction to the actin skeleton. Below the tight junctions are adherens junctions composed of transmembrane proteins, E-cadherin and nectin linked to the cytoskeleton by scaffolding proteins, catenin and afadin linked to actin filaments. Desmosomes are made up of the transmembrane liner glycoproteins, desmoglein and democollin which are cadherin proteins linked to intermediate keratin filaments. Gap junctions form a tunnel for small molecules to pass between adjacent epithelial cells. Image made using BioRender.

An important component of the intestinal barrier is the ability of the single layer of intestinal epithelial cells to attach to each other by forming intracellular junctional complexes, composed of tight junctions, adherens junctions, desmosomes, and gap junctions. Tight junctions are critical for maintaining the physical barrier integrity by selectively limiting the diffusion of small molecules, water, and ions as well as protecting the body against infection and inflammation. The integrity of tight junctions is regulated by the arrangement of actin together with the interaction between the peripheral and integral transmembrane proteins such as occludins, claudins, and junctional adhesion molecule (JAM) to form a tight seal between adjacent epithelial cells and are at the apical part of the junctional complex. The complex is strengthened by cytoplasmic scaffolding and adapter proteins, such as ZO-1, -2, and -3 (Zona occluden) (**Figure 4**) (31). Tight junctions act as a fluid barrier preventing the diffusion of solutes, ions, and other molecules across between the two cells. Thus, abnormalities in tight junctions are associated with metabolic and inflammatory diseases, as well as cancer, since tight junctions can be affected by pathogens and commensal bacteria (32–35). Below the tight junctions are the adherens junctions important for cell to cell signaling by joining the actin filaments of neighboring epithelial cells and supporting epithelial stability (36). Adherens junctions consist of E-cadherin and nectin transmembrane spanning proteins and these proteins are anchored by intracellular components of the catenin family, β-catenin which binds α-catenin, which in turn bind to other proteins such as afadin in a protein complex with actin filaments, regulating the organization of the actin cytoskeleton (31, 37, 38). The extracellular domain of the nectin transmembrane protein bind to those nectin on neighboring epithelial cells, while the cytoplasmic tail binds afadin (**Figure 4**). At the basolateral end of the epithelial cells are desmosomes and these cannot prevent the movement of solutes between cells as they are not continuous, instead they mainly provide mechanical strength by joining the intermediate filaments of neighboring epithelial cells involved in cell-cell adhesion (35, 39). The cytoplasmic side of each plasma membrane has a plaque belonging to the plakoglobin (Pg) and plakophilins or the plakin family (desmoplakin), and these are joined to cells by linker proteins which includes the cadherin proteins, desmoglein, and desmocollin in the extracellular space (40). The intermediate keratin filaments anchor the desmosomes together at the opposite sides of the cells by attaching to the cytoplasmic protein plaque (**Figure 4**). Gap junctions are made up of connexins that form a channel to allow the transport of small molecules between neighbouring cells and play a regulatory role in cell growth and differentiation (**Figure 4**) (41).

### Regulation of Tight Junctions

The assembly and maintenance of tight junctions are regulated by signaling pathways, such as protein kinases C (PKC) and myosin light chain kinases (MLCK). ZO-1 and occludin interact with PKC and phosphorylation of tight junction proteins by PKC is associated with tight junction regulation. As the C-terminus of occludin interacts with ZO-1 and is phosphorylated on the serine/threonine residues (42). Once stimulated, the tight junctions open leading to the paracellular movement of ions, solutes, and peptides across the intestinal epithelium (26). Proinflammatory cytokines such as TNF-α and IFNγ also cause an increase in tight junction permeability, since TNF-α activates the NF-κB pathway and decreases the level of ZO-1 protein with IFNγ also causing a reduction in ZO-1, both suppressing the tight junction barrier function (43, 44). TNF-α stimulation of NF-κB signal transduction pathway regulates MLCK, increased levels of MLCK leads to an increase in tight junction permeability. This allows LPS to leak into the bloodstream leading to low-grade inflammation, including steatosis and insulin resistance. As LPS can interact with immune cells and adipocytes, this then drives a potentially chronic, systemic inflammation. Inhibition of the activation of NF-κB by pharmacologic inhibitors cause the inhibition of the activation of MLCK and leads to a decrease in tight junction permeability (45).

**Figure 5:**
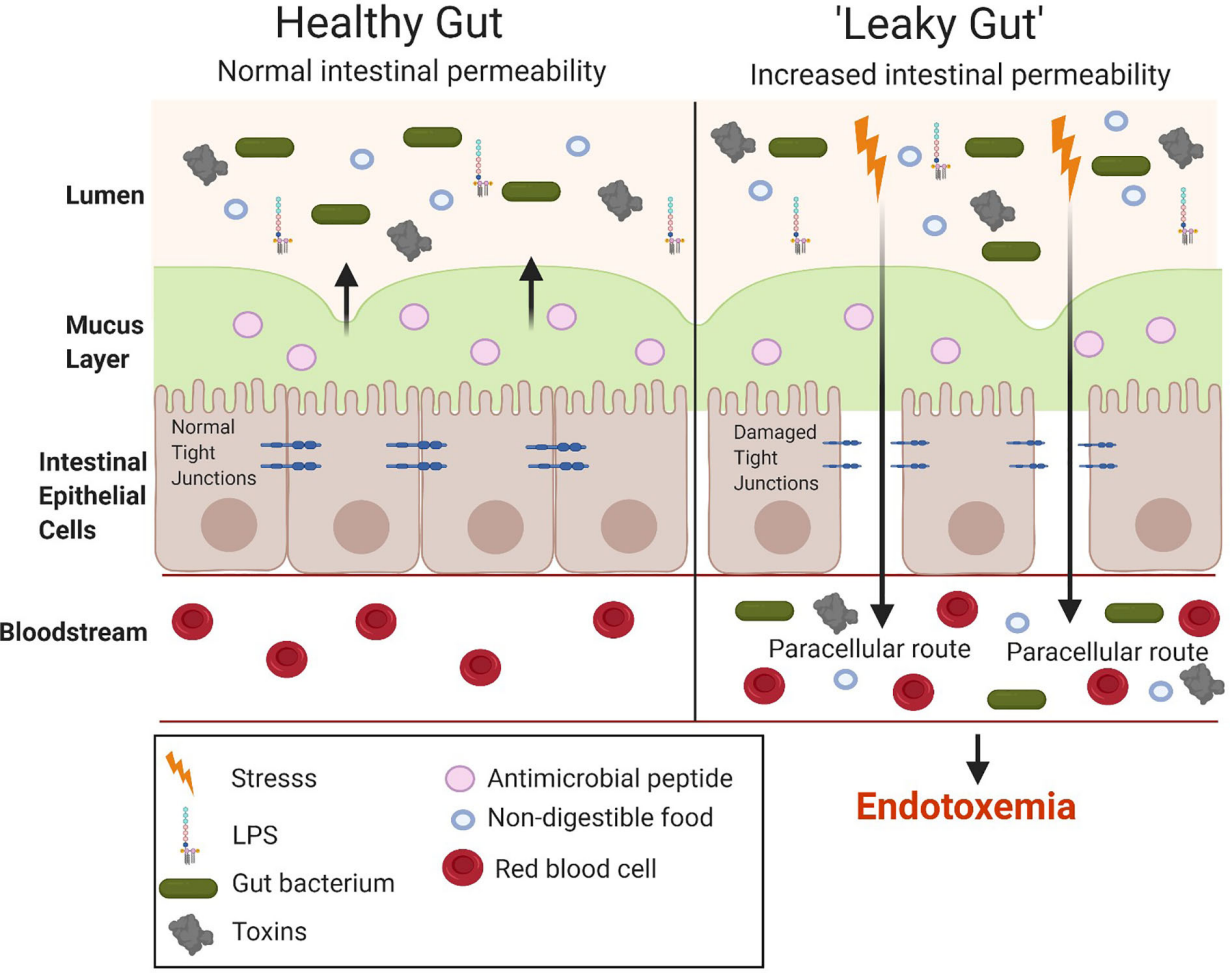
Comparison between a healthy and “leaky gut.” A “leaky gut” affects the lining of the intestine and tight junctions are damaged in response to stress for example, a high-fat diet, this allows the passage of LPS and other pathogens into the bloodstream, causing the activation of the immune system and inflammation. Image made using BioRender.

The small intestine is important for the absorption of nutrients and, hence, has a larger surface area than the large intestine to allow the digestion and absorption of nutrients. However, the large intestine has a higher number of bacteria (10^11^) compared to the small intestine (10^8^) (46). Hence, although the small intestine is the easiest for the translocation of LPS it does not have the highest bacterial concentration which is seen in the large intestine. The gut microbiota is considered the main source of the LPS in metabolic endotoxemia. The number of gut bacteria is approximately 10-times that of all the cells present in the human body (47). LPS alters the intestinal epithelial tight junction protein assembly (ZO-1 and occludins) of the intestinal epithelium contributing to a “leaky gut,” allowing the translocation of LPS from the lumen of the digestive tract into the bloodstream, resulting in endotoxemia and the overproduction of pro-inflammatory cytokines (**Figure 5**). Tight junction permeability is mediated by TLR-4 activation by MyD88 on the membrane of the epithelium, allowing the intestinal epithelium to respond to inflammation by bacteria and proinflammatory cytokines, ultimately leading to intestinal inflammation (26). Many studies have investigated the intestinal bacteria as a main source of LPS, ultimately causing metabolic endotoxemia (after translocation to the luminal side) (48–50). While the cytokine storm triggered by the release of large amounts of LPS (sepsis) can lead to shock and potentially organ failure, the long term effects of low-grade endotoxemia are associated with an increased risk of cardiovascular disease and T2DM driven by metainflammation (51, 52).

Metabolic and inflammatory disorders including obesity, non-alcoholic steatohepatitis (NASH), and inflammatory bowel disease (IBD) are linked to a defect in tight junction barrier of the intestinal epithelial. Therefore, maintaining tight junction integrity is a possible strategy to treat these diseases. HFD induced metabolic disorders are associated with gut dysbiosis in both animal and human studies have investigated the effect of various HFD on the alterations of the microbiome within the intestine (53, 54). HFD is also associated with alterations in the intestinal barrier, which allow an increase in permeability and hence, the translocation of LPS into the bloodstream activating the TLR4 signaling pathway (49, 55).

## Evidence of Metabolic Endotoxemia in Human and Animal Studies

Metabolic endotoxemia causes both local and systemic inflammation, which contributes to many cardiometabolic diseases associated with obesity (51). There has been convincing evidence for metabolic endotoxemia in both human and animal studies.

When comparing 559 overweight males and females with 500 volunteers of normal weight, LBP was elevated (assumed to be secondary to endotoxemia). LBP is a marker of the acute phase response, hence, serum LPS levels frequently correlate with conditions associated with low-grade inflammation. Thus, it is difficult to conclude with certainty whether a rise in LBP in diseases associated with inflammation is secondary to the systemic inflammation or due to endotoxemia (56). Nevertheless, plasma LBP levels have been investigated in many metabolic disorders, as LBP is synthesized in the liver and initiates the recognition of LPS, delivering LPS to other adaptor molecules to trigger the TLR4 signaling pathway and the production of proinflammatory cytokines. Plasma LPS levels were 76% higher in patients with T2DM when compared to control participants. In obesity and T2DM, LPS also activates an immune response in human adipose tissue. In patients with T2DM, the activation of NF-κB results in the formation of adipocytokines in adipose tissue. In T2DM, the function of Kupffer cells is impaired, resulting in a reduction in the clearance of LPS. Treatment of human abdominal subcutaneous adipocytes with LPS resulted in a significant increase in the secretion of the pro-inflammatory cytokines, TNF-α and IL-6. In addition, the expression of TRAF6 and MyD88 is increased in patients with T2DM. Inhibition of the activation of NF-κB in human abdominal subcutaneous adipocytes reduced the formation of IL-6. Taken together, these findings suggest that T2DM is associated with endotoxemia, which leads to the activation of abdominal, subcutaneous adipocytes and a subsequent immune response (57). In 192 individuals with atherosclerosis, the serum levels of endotoxin were positively correlated with the waist to hip ratio, waist circumference, serum insulin, triglycerides levels, and total cholesterol. However, endotoxin was negatively correlated with serum HDL-cholesterol (58). In children with NAFLD, the serum endotoxin, TNF-α, IL-6, and PAI-1 levels were higher suggesting that endotoxemia can contribute to the progression of NAFLD (59). In human studies, a controlled-feeding study showed within 24 h of initiating a high-fat/low fiber or low-fat/high fiber diet there was a positive association between *Bacteroidetes* and *Actinobacteria* in fat and a negative association between *Firmicutes* and *Proteobacteria* (60).

Metabolic syndrome is a group of conditions that occur together, leading to an increased risk of cardiovascular risk factors. Tissue factor (TF) in the circulation triggers the procoagulant pathway of coagulation and in atherosclerotic plaques they are expressed on peripheral blood monocytes (PBMCs). Stimulation of PBMCs with LPS leads to the induction of monocyte TF and an upregulation of LPS in patients with metabolic syndrome (61). Therefore, suggesting metabolic syndrome upregulation of monocyte TF is significantly associated with low-grade inflammation.

Numerous studies have suggested a link between high fat-or high-energy, high carbohydrate diets and endotoxemia. HFD can cause changes to bacterial diversity, increased permeability and integrity of gut wall membrane (62). A population-based study of 1,015 healthy men reported a link between energy intake and the plasma levels of LPS, and a similar link was found in mice challenged with a high fat-or high-energy, high carbohydrate diet for 4 weeks (63). Many other studies have found a link between a high fat-or high-energy, high carbohydrate diet and an increase in LPS (64, 65) also an increase in the expression of TLR2 and TLR4 (64). Mouse models have shown the importance of TLR4 and its signaling in diet-induced insulin resistance and atherosclerosis (66). TLR4 knockout mice challenged with a HFD develop less insulin resistance and have a better glucose tolerance test than their wild-type litter mates (67, 68). TLR4-deficient mice also develop less atherosclerosis (69). Specifically, atherosclerosis-prone ApoE^−/−^ mice deficient in TLR4 or MyD88 have less atherosclerotic plaques associated with a reduction in macrophage recruitment (70). An increase in serum LPS was also observed using the limulus amebocyte lysate assay when investigating the effect of nutritional intake in patients with cardiometabolic diseases, supporting the role of bacterial infections and the immune response in cardiometabolic diseases (52). In 208 healthy male subjects over 4 weeks consuming a high-fat or high-carbohydrate diet showed a link between food intake and the levels of plasma LPS (63).

Many studies have looked into the mechanism(s) by which HFD can cause the translocation of LPS: HFD causes an increase in the gut permeability and/or changes in the composition of gut microbiota, which in turn result in the formation of larger amounts of LPS which then translocate into the bloodstream. Feeding mice a HFD for 4 weeks resulted in an increase in serum LPS (metabolic endotoxemia) due to changes in gut microbiota: Specifically, HFD caused a reduction in the intestinal bacteria, *Bifidobacterium* and *Eubaterium* spp (1).. This study triggered the interest in HFD-induced dysbiosis and raised questions about the importance of the intestinal microbiota in obesity and related comorbidities. In another study, exposure of mice to a HFD for 7 weeks caused an increase in body weight, blood glucose, higher levels of hepatic triglycerides, and a reduction in the ratio of *Bacteroidetes* to *Firmicutes* (71). In rats, a HFD for 16 weeks resulted in an increase in the intestinal permeability, a reduction in the expression of tight junction proteins (claudin-1, claudin-3, occludin, and junctional adhesion molecule-1) in the small intestine and an increase in plasma TNF-α (72). Feeding mice a HFD for 4 weeks caused an increase in the intestinal permeability and a reduction in the tight junction gene expression, suggesting that HFD has an effect on intestinal function (55). Interestingly, genetically obese mice (ob/ob and db/db) also have elevated levels of LPS and pro-inflammatory cytokines IL-1β, INFγ, and TNF-α (73). These findings support the view that an increase in intestinal permeability drives systemic endotoxemia. Other animal studies showed that feeding mice a HFD for over 8 weeks resulted in an increase in LBP. Mice lacking TLR4 or the co-receptor CD14 showed no change in body weight (74).

## Potential Pharmacological Interventions

Multidrug-resistant Gram-negative bacteria is becoming an increasing problem. Hence, new strategies are needed to overcome this and protect patients. The activities of LPS are mediated by the lipid-A residue of the molecule, containing the molecular components which is important to determine the endotoxic activity of LPS. Therefore, anti-endotoxin therapies have been considered as potential treatment strategy. Antibodies are effective for therapeutics due to their specificity. For example, O-polysaccharide specific antibodies reduce the toxic effects of LPS. In the 1960’s, more research was conducted regarding the structure of LPS and it was made clear that the O-polysaccharide part of LPS was immunodominant, as immunizing animals with specific stereotypes of bacteria led to the induction of antibody production directed against the specific O-polysaccharide (75). Another possible intervention includes approaches to neutralize the toxicity of the lipid A moiety of LPS, hence preventing the interaction with host tissues such as polymyxins. Polymyxins are antibiotics that combine a bactericidal activity with the ability of neutralizing endotoxins (76). Due to the excessive use and misuse of antibiotics, there is increasing antibiotic resistance which is becoming a major problem. Antibiotic resistance places an economic burden on the health care system and each year about 700,000 people worldwide die from drug-resistant strains (77). Therefore, antibiotic alternatives are needed to reduce the number of deaths and to allow a reduction in antibiotic use.

### Antimicrobial Peptides

#### Mechanism of Action of Antimicrobial Peptides

Even though AMPs are part of innate immunity, the mechanism of action of killing microbes differs from those of phagocytes and cytokines. AMPs are capable of killing bacteria by interacting with bacterial cell wall or membranes (78). Most AMPs are induced in response to cytokines or PAMPs and are synthesized shortly after an infection to neutralize a range of microbes, while some are constitutively expressed and are stored as inactive precursors in granules within phagocytes at high concentrations and protect their hosts against many microorganisms as they are locally released at sites of infection and inflammation (79). They have a broad spectrum and can target organisms such as fungi, yeast, viruses, and bacteria (Gram-positive and Gram-negative). AMPs also prevent the formation of biofilms and they have anti-cancer properties (80).

The mechanism by which AMPs bring about their antimicrobial effects are not entirely understood but include direct killing and immune modulation. These mechanisms are dependent on several physicochemical properties of the AMP such as secondary structure, charge, and amphipathic properties. All biological membranes are composed of a fluid mosaic, made up of phospholipids and proteins. Therefore, AMPs with ~50% hydrophobic residues fold into a amphipathic confirmation and with a net positive charge (+2 to +9) (81), allows the peptide to interact with the negatively charged bacterial phospholipid head groups on the inner leaflet of the membrane, disrupting the bacterial cell membrane non-enzymatically and allowing the translocation of the peptide into the bacterium to affect internal targets (80).

Microbial membranes differ from animal cell membranes and this allows AMPs to distinguish their target, as they are more selective for bacterial membrane, since animal cell membranes have zwitterionic rather than negatively charged phospholipids and also contain cholesterol that helps to protect the membrane from damage (81). Therefore, there is only a weak interaction with the zwitterionic phospholipids of plant and mammalian membranes. The electrostatic interaction of the peptide with the negatively charged molecules on the bacterial membrane is a strong interaction and this causes disruption, such as pore formation in the bacterial membrane leading to the destruction of the integrity of the membrane. It is this interaction that is the main mechanism of action of AMPs for producing their antimicrobial activity. Studies have looked at the correlation between charge and the antimicrobial activity of AMPs, a charge of +3 to +5 showed to improve the antimicrobial activity of the AMP against both Gram-positive and Gram-negative bacteria. However, increasing the charge to +6 or +7 caused a loss in the antimicrobial activity, suggesting too strong of an interaction and hence preventing the peptide from translocation (80). This suggests the importance of charge in the mechanism of action of AMPs.

In other cases, AMPs produce their antimicrobial activity by entering the bacterium by translocation to inhibit intracellular functions such as nucleic acid synthesis, protein synthesis, and enzymatic activity. Both mechanisms of action of AMPs lead to bacterial lysis (78). However, some peptides can cross the lipid barrier without causing damage to the bacterial membrane, such as by blocking the enzyme activity or inhibiting the synthesis of proteins. For example, phospholipase A_2_ (PLA_2_) is secreted from platelets and hydrolyses the bacterial membrane by enzymatic digestion to kill the bacteria (78).

Some AMPs modulate host immunity by either activating or recruiting immune cells of the adaptive immunity such as, T-cells, macrophages and neutrophils or by effecting toll-like receptor responses, thereby controlling inflammation and/or increasing bacteria clearance (82). The human cathelicidin, LL-37, has been shown to act as a chemoattractant, attracting neutrophils, mast cells, monocytes, and T-cells. This is achieved by LL-37 acting as a ligand of the formyl peptide receptor-like 1 (FPRL1) and the G_i_ coupled receptor (83). AMPs have also shown to have anticancer activity, as the outer layer of cancer cells is often a negatively charged phosphatidylserine (PS), hence enhancing the interaction with AMPs (84). AMPs are also able to specifically target cancer cells, as these overexpress of other negatively charged molecules, such as O-glycosylated mucins and heparan sulphate (80).

The rising rate of multi-drug-resistant bacteria has led to difficulty in treating patients that are critically ill due to the misuse or excessive use of antibiotics. Hence, AMPs have become a focus as new treatments of infections. Killing of bacteria by antibiotics results in the release bacteria-derived wall fragments, including LPS or lipoprotein (LP). This leads to the initiation of the pro-inflammatory cascade potentially causing harm to patients taking these anti-infective agents (85). Understanding the importance of AMPs in the gut could lead to the discovery of novel therapies for enteric infection, since antimicrobial peptides not only scavenges the LPS that is released when they kill bacteria but they also manage microbiome diversity (86).

#### Intestinal Antimicrobial Peptides

The human gut is colonized with trillions of microorganisms and the small intestine is the main site for microbial colonization. AMPs including C-type lectins and α-defensins, lysozymes, and phospholipase A2 are secreted by Paneth cells, located at the bottom of crypts of *Lieberkuhn* to protect against infections and help to maintain the intestinal homeostasis (87). Intestinal AMPs are capable of ingesting pathogens and play a role in maintaining a healthy balance between the host and the number of commensals (78).

#### Defensins

The largest group of AMPs, the defensins, are involved in antiviral, antibacterial, immune, antifungal, and inflammatory responses and are expressed in both vertebrates and invertebrates. Defensins are an important part of the innate immunity and have β-sheet structures and are cationic peptides with antimicrobial properties. Humans have 17 defensins, all of which consist of a β-sheet fold stabilized by three disulfide bonds formed by six conserved cysteine residues and are classified into three different subfamilies based on the location of the disulfide bond: α, β, and θ defensins (88, 89). The human α-defensins 1–4 (HD1–4) is also known as the human neutrophil peptides (HNP1–3), stored in the granules of neutrophils and are found in the airways. In particular, HBD-3 has shown to reduce LPS induced TNF-α and IL-6 in both mouse and human macrophages (90). Human β-defensins 1–4 (HBD 1–4) are secreted predominately by epithelial cells and in humans there are six enteric defensin α-defensins, DEFA1–6 (91). Human β-defensin 5 and 6 (HD5 and 6) are both produced by Paneth cells and five mouse β-defensins (mBDs) (88, 91).

Enteric α-defensins are an important component of the mucosal innate immunity which was first discovered in Paneth cells in mouse small intestine which are stored in secretory granules as the inactive pro-peptide and a proteolytically cleaved to allow for their antimicrobial activity (92). Paneth cells contain a matrix metalloproteinase enzyme, matrilysin (MMP-7), which is important for processing the active form of the peptide (93). Whereas, HD-5 and HD-5 are processed by trypsin following their release. The importance of MMP-7 was shown in MMP-7 knockout mice, the MMP-7 deficient mice only secreted the inactive precursor form of the propetide in their Paneth cells (94). In the ileum of the small intestine, LPS causes the activation of MMP-7 and MMP-7 knockout mice are protected against LPS-induced leakage of the gut during endotoxemia. Hence, MMP-7 knockout mice show less severe inflammation in the gut with a reduction in the production of TNF and IL-6 in the ileum (95). Patients with Crohn’s disease have a decreased expression of α-defensins, suggesting that AMPs reduce enteric inflammation and/or prevent the development of inflammatory bowel disease (96).

Defensins could be a potential intervention used for the treatment of endotoxemia, as they play a key role in the innate immune system. Human defensins are naturally occurring, non-toxic microbicides that protect the host from invading microbes in the small intestine. Human beta defensin 1 (hBD1) has minor antibiotic activity, however, reducing the disulfide bridges in hBD1 increases the antimicrobial activity (97). hBD1 is secreted as an oxidized form of the peptide, HD5_OX_, which has the widest spectrum of antibacterial activity of the α-defensins. Thus, HD5_OX_ could be the most promising target against antibiotic resistant pathogens. HD5_OX_ causes changes to *Escherichia coli* and other Gram-negative microbes. This morphological change includes bleb formation and a study using *E. coli* expressing GFP showed that treatment with HD5_OX_ caused GFP emission in the bleb (98).

#### Cathelicidin

All members of the cathelicidin family of peptides have a conserved amino-terminal cathepsin L inhibitor domain (cathelin) and play an essential role in host defense (99). Cathelicidins have an α-helix structure with a highly conserved N-terminal region and a C-terminal domain, which contains the antimicrobial properties. Cathelicidin is produced by several cell types such as keratinocytes, immune cells, urinary tract, sebocytes, and bone-marrow-derived cells found within the skin (100). The first mamalian, cathelicidin AMPs were isolated from bovine neutrophils and named Bac5 and 7 (101). Cathelicidins are one of the most diverse AMPs of vertebrates and are found in many mammals including sheep, bovines, horses, goat, and mice (80).

In addition to the antimicrobial properties of AMPs, LL-37/hCAP18 is the only member of the cathelicidin family of AMPs known in man and it regulates inflammatory and immune responses. In humans, cathelicidin antimicrobial peptide (CAMP) encodes for LL-37, which has also been named, hCAP18 which is the precursor protein. LL-37 contains a signal peptide, N-terminal domain and a C-terminal domain. The C-terminal domain has a variety of activities and is called LL-37, as the protein releases LL-37 from its C-terminal domain. LL-37 is a 37-amino acid residue, α-helical peptide and is amphipathic. LL-37 is found throughout the body, but is mainly produced in mast cells, neutrophils and monocytes (102). LL-37 has bactericidal properties and are capable of neutralizing pathogen factors, such as lipopolysaccharides (LPS) or lipoprotein (LP), both of which are released during infection/injury. Through electrostatic interactions, LL-37 can bind to bacterial membranes and cause membrane disruption and hence peptide insertion (78). A study showed that LL-37 treated with *E.* *coli* demonstrated bacterial lysis properties using electron microscopy and that LL-37 is protected against the action of proteolytic degradation, since in solution it forms aggregates and it also forms a lipid bilayer (103, 104).

As the human AMP, LL-37 is toxic at high doses, synthetic (mimetics) AMPs were developed incorporating a specific sequence structure to develop synthetic anti-lipopolysaccharide peptides (SALPs) with an improved ability to bind and neutralize the lipid A moiety of LPS in order to reduce toxicity and optimize antimicrobial activity. Peptide 19-2.5 belongs to the new synthetic AMPs and has been designed to strongly bind LPS and causes the lipid A part of LPS to convert from a cubic aggregate into an inactive multi-lamellar structure and has efficacy in preclinical models of sepsis (105). There is good evidence that Peptide19-2.5 attenuates the systemic inflammation and organ injury/dysfunction associated with sepsis by binding to and inactivating LPS (106). Peptide 19-2.5 attenuates septic cardiomyopathy and prevents the downregulation of SERCA2, which plays a pivotal role in the cardiac dysfunction associated with sepsis (106). This peptide combined to several unrelated antibiotics neutralize the serum levels of TNF-α to control endotoxemia induced by bacterial infection in mice (107). Hence, Peptide 19-2.5 can act as an anti-endotoxin, since this peptide can effectively kill both Gram-negative and Gram-positive bacteria, as well as being able to neutralize endotoxin by sequestering LPS (107). Therefore, suggesting a combination therapy of AMPs with antibiotics may be an effective treatment for endotoxemia. Thus, synthetic AMPs, such as Peptide 19-2.5 could be a potential lead candidate for the treatment of metabolic endotoxemia.

## Potential Dietary Interventions

Treatment of metabolic endotoxemia is vital due to the range of chronic diseases related to endotoxemia. Diet-based interventions targeting the gut microbiota could reduce circulating levels of endotoxins, hence can prevent the development of chronic low-grade inflammation associated with endotoxemia. Modifying lifestyle including minimizing alcohol consumption, increasing diet rich in macronutrients, and reducing saturated fat intake can all have an impact on reducing metabolic endotoxemia, as well as prebiotics and probiotics have shown to reduce the levels of circulating endotoxins.

Consuming a large amount of alcohol can cause changes to the gastrointestinal tract since the enzyme for oxidative metabolism of alcohol is present in the intestinal mucosa and the liver becomes overwhelmed, leading to the (i) disruption of the intestinal barrier integrity causing an increase in the permeability hence, elevating endotoxin levels in the systemic circulation, (ii) altering the intestinal microbiota resulting in the production of LPS (108). A positive correlation was found between the quantity of alcohol consumption and the serum LPS levels (109). These high endotoxin levels are due to the prevention of Kupffer cells effectively clearing these molecules from the circulation (110). The main oxidative metabolite in alcohol, acetaldehyde alters the structure of the intestinal barrier by disrupting the epithelial tight junctions therefore causing an increase in intestinal permeability (111). Alcohol feeding in mice results in an increase in the liver injury marker, serum alanine aminotransferase (ALT), triglycerides and steatosis. However, this was not seen in TLR4-KO mice (112). This suggests the importance of the activation of TLR4 receptors by LPS on Kupffer cells in liver injury.

Micronutrients are important in altering the gut microbiota and micronutrient deficiencies affect the composition of the gut microbiota. A diet lacking one or all of the four micronutrients investigated: vitamin A, zinc, iron, or folic acid disrupted the composition of the gut microbiota, with vitamin A deficiency having the largest effect on altering the composition resulting in an increase in *Bacteroides vulgatus* (113). Zinc is important in maintaining the structure and function of the membrane barrier, hence depletion of zinc was found to affect the barrier integrity and function and cause an upregulation of chemokines (114). Vitamin D is also important as treatment found to increase the levels of claudin-1, claudin-2, and ZO-1, however mice lacking vitamin D receptor, showed severe disruption in the epithelial junctions after 3 days following dextran sulphate sodium treatment to induce colitis (115). Hence micronutrient deficiencies lead to an increase in intestinal permeability and ultimately metabolic endotoaemia.

Diets high in saturated fats contribute to metabolic endotoxemia and unsaturated diets such as the Mediterranean diet rich in fruits, vegetables, nuts, and whole grain reduce metabolic endotoxemia (116). Oils rich in saturated fats including the western diet: vegetable, palm, and canola oils cause endotoxemia, compared to oils rich in n-3 polyunsaturated fatty acids (PUFA) attenuate endotoxemia. Also, dietary cod liver and fish oils attenuate the level of endotoxin concentration compared to coconut oil (117). The structure of fat that is consumed leads to the changes in composition, hence altering the extent of endotoxemia. Food additives including sugar, surfactants, and sodium chloride, which are applied in high concentrations to commonly consumed foods, have been suggested to increase intestinal permeability (118, 119). A Drosophila model and a human cell co-culture model was used to demonstrate the effect of additives on the function of the gut barrier, showing an increase in intestinal permeability (119).

Metabolic endotoxemia has been implicated in gut dysbiosis, hence probiotics have been investigated as a dietary strategy. The gut microbiota is the product of the interaction between the hosts genetics and environment factors such as diet which influences bacterial population. Gut microbiota is an important determinant of metabolic disorders such as obesity and T2DM, since western diets have been shown to alter the bacterial population causing the activation of proinflammatory mechanisms and disrupting the intestinal barrier, hence leading to metabolic endotoxemia (120). Therefore, manipulating the gut microbiota with probiotics could be used as a potential intervention to manage metabolic diseases. Probiotics are defined as live microorganisms and the main members of this group include, *Bifidobacteria* and lactobacilli (120, 121). Orally administered probiotics, microencapsulated *Bifidobacterum infantis* in rats for 38 days showed a significant increase in *Bifidobacteria* and reduced serum endotoxins (122). HFD-fed mice received *Bifidobacteria* for 4 weeks, this resulted in a reduction in weight gain and attenuated the increase in blood glucose, triglycerides, and total cholesterol caused by a HFD (123). Probiotics change the bacterial composition in the gut as they are in competition for metabolites, antimicrobial proteins, and nutrients. Prebiotics also modulates the gut microbiota and increases bifidobacterial. The main known prebiotics are non-digestible carbohydrates such as inulin and fructooligosaccharides (FOS) (124). Studies have shown the beneficial effects of prebiotics for reducing metabolic endotoxemia (125). A clinical study with 30 obese women receiving maltidextrin or insulin-type fructans prebiotics daily for 3 months showed a significant increase in *Bifidobacterium* with *Bifidobacterium longum* showing a negative correlation with the levels of serum LPS (126). Thus, dietary interventions could control the level of metabolic endotoxemia.

## Limitations

### LAL Assay

The LAL assay is commonly used to detect endotoxins, however, there are several limitations of this assay when aiming to reliably quantify LPS levels in the blood. Firstly, there are limitations in collecting blood samples to measure LPS in animals, as the blood would have to be taken from the portal vein, which is not a trivial procedure. It is also essential to avoid the contamination of the blood sample with LPS during sample collection. There is evidence that heparinized blood collection tubes are frequently contaminated with endotoxin and this may lead to a false positive result (127). False positive results may also be secondary to the presence of plasma lipids, as these increase the sensitivity of LAL assay due to the direct activation of LAL in samples in which either triglycerides or very low-density lipoproteins concentrations are high. Thus, blood samples taken from subjects with T2DM, which frequently have high serum triglyceride levels, have a higher chance to deliver false positive results (128). To overcome this limitation, LPS and mannan can by extracted in the nonlipid phase (129). It should be noted that the LAL assay is also not endotoxin specific, as the assay can also be activated by β-glycans, peptidoglycan from Gram-positive bacteria, simple polysaccharides and dithiols (130, 131). False negative results are also possible for the following reasons: The lipid A component of LPS associates with the phospholipid shell of serum lipoproteins when entering the blood stream and this may hinder the detection of LPS by the enzymes of the LAL assay (132). In addition, the half-life of LPS is very short, as it is rapidly sequestered by LBP (133). To overcome all of these limitations (which is challenging), one could use several assays togethers with the LAL assay, such as the LBP assay and the endotoxin activity assay (EAA) (134). Thus, care should be taken when attributing any pathology associated with a positive LAL test result to the presence of LPS (cause-effect-relationship). Further studies are necessary to clarify the role of LPS in the pathophysiology of diseases associated with metainflammation. Another limitation of the LAL assay is suggested since the measurements of serum levels of LPS in healthy individuals have shown to differ in different studies ranging from 0.04 to 0.36 EU/ml (133). Also, the sample preparation method used changes the levels, when samples are treated with perchloric acid or Tween 80 the levels are higher compared to heating and dilution (133).

### Interventions Using AMPs

The gut microbiota confers health benefits to the host by aiding both the protection of the gut and nutrient absorption (135). Thus, the non-specific killing of bacteria in the gut by AMPs may have detrimental effects on gut health. However, AMPs kill bacteria at non-toxic concentrations (136). AMPs are becoming a focus as new treatments, as the rising rate of multi-resistant bacteria, poses a challenge for the treatment of patients with severe infections, due to either misuse or excessive use of antibiotics. There is also the added problem that antibiotics kill bacteria; however, they also cause the release of bacterial wall fragments including lipopolysaccharide (LPS) or lipoprotein (LP). This leads to the initiation of the pro-inflammatory cascade and this can potentially cause harm (85). Therefore, there is a need for new treatments that are both effective and safe. The World Health Organization has stated antibiotic resistance as one of the biggest threats to human health (137). By 2050, it has been estimated that 10 million people worldwide will die every year from drug-resistant bacteria (138). AMPs could potentially be used as new treatments, as they kill bacteria, fungi, and viruses. The antimicrobial properties of AMPs are produced in many ways, as they have multifunctional properties, and this makes them less likely to become resistant to microorganisms. A combination therapy of both antimicrobial peptides and antibiotics may be a promising approach, which would allow a reduction of the doses of antibiotics, therefore lowering the potential side-effects, while the combination therapy may also prevent the development of bacterial resistance. However, any long-term treatment with AMPs may also trigger the development of resistance and the rate is determined by several factors, such as the mutation supply rate (139). AMPs can develop mechanisms to resist the action of antibiotic in their natural environment of bacteria in intrinsic resistance and acquired resistance is due to mutated genes that allow bacteria to grow when AMPs are present (136). Resistance can also occur due to changes in the net surface charge of the bacteria, hence reducing their attraction to AMPs (81). Other mechanisms of AMP resistance includes the proteolytic degradation of AMPs by extracellular proteases of Gram-positive bacteria or electrostatic repulsion by aminoacylated peptidoglycans of Gram-negative bacteria (140).

### Antibody Therapy

Antibody therapies as a potential intervention for metabolic endotoxemia has limitations, since there is diversity in O-polysaccharide targets that are expressed by different Gram-negative bacteria in the gut. Antibody specificities are directed mainly against the O-polysaccharide of LPS, hence are specific for one particular O-antigen. Therefore, this makes it not possible to use one anti-LPS antibody to target one bacterium.

## Conclusion

The concept of metabolic endotoxemia is still controversial, as most of the evidence in support of a role of endotoxemia as the key driver of meta-inflammation relies on data generated with the LAL assay, which has significant limitations. Findings indicating that TLR-4 knockout mice have less pathology when challenged with a HFD would support the notion that metabolic endotoxemia plays a key role in the pathophysiology. Similarly, the finding that ApoE-knockout mice deficient in TLR-4 have less atherosclerotic burden also supports the notion of a role of metabolic endotoxemia in atherosclerosis. The question of a true cause-effect relationship of LPS in metabolic disorders will have to be addressed by pharmacological or dietary interventions that specifically target metabolic endotoxemia and this remains a significant challenge.

## Author Contributions

SM wrote the manuscript. CT proofed and advised on the content of the manuscript. All authors contributed to the article and approved the submitted version.

## Funding

SM is supported by the British Heart Foundation (BHF) MRes/PhD Scholarship (Award number: FS/17/69/33484).

## Conflict of Interest

The authors declare that the research was conducted in the absence of any commercial or financial relationships that could be construed as a potential conflict of interest.
